# Ergodicity Breaking and Self-Destruction of Cancer Cells by Induced Genome Chaos

**DOI:** 10.3390/e26010037

**Published:** 2023-12-29

**Authors:** Sergey Shityakov, Viacheslav Kravtsov, Ekaterina V. Skorb, Michael Nosonovsky

**Affiliations:** 1 Infochemistry Scientific Center (ISC), ITMO University, 9 Lomonosova St., 191002 St. Petersburg, Russia; skorb@itmo.ru; 2 College of Engineering and Applied Science, University of Wisconsin-Milwaukee, Milwaukee, WI 53211, USA

**Keywords:** genome chaos, cancer, ergodicity, BCL2, mutation rate, chromothripsis, rhabdomyosarcoma

## Abstract

During the progression of some cancer cells, the degree of genome instability may increase, leading to genome chaos in populations of malignant cells. While normally chaos is associated with ergodicity, i.e., the state when the time averages of relevant parameters are equal to their phase space averages, the situation with cancer propagation is more complex. Chromothripsis, a catastrophic massive genomic rearrangement, is observed in many types of cancer, leading to increased mutation rates. We present an entropic model of genome chaos and ergodicity and experimental evidence that increasing the degree of chaos beyond the non-ergodic threshold may lead to the self-destruction of some tumor cells. We study time and population averages of chromothripsis frequency in cloned rhabdomyosarcomas from rat stem cells. Clones with frequency above 10% result in cell apoptosis, possibly due to mutations in the BCL2 gene. Potentially, this can be used for suppressing cancer cells by shifting them into a non-ergodic proliferation regime.

## 1. Introduction

The concept of ergodicity plays a central role in many problems of dynamical systems. Ergodicity is defined as the equivalence of time averages and phase space (or ensemble) averages in a physical system. In addition to its theoretical significance, ergodicity is crucial for practical aspects of measuring the system’s parameters, since sufficiently long observations of temporal behavior are often impossible, and therefore, it is desirable to substitute them with finite time measurements [1,2,3]. In biophysical and biomedical applications, ergodicity is particularly important for processes that may involve chaotic behavior. These processes include the transport of blood and other liquids, intra- and extracellular fluid flow in cytoplasm and nucleoplasm, and multiphase flow involving macromolecular biopolymer solutions [3,4,5,6].

Typically, the chaotic behavior implies that a dynamic system is ergodic, since the information about the system’s history is constantly erased due to growing fluctuations. However, the interrelation between chaos, turbulence, and ergodicity is often quite complex. Experimental simulations have suggested that turbulent chaotic behavior governed by the Navier–Stokes equation is ergodic [7]. Despite that, many biological systems are non-ergodic. The causes of ergodicity breaking are diverse. Many of them simply lead to the anomalous diffusion deviating from the classical linear Einstein–von Smoluchowski dependency of the mean-square displacement on the lag time. Such causes include macromolecular crowding, aging-induced flow through obstacles, and the so-called “hydrodynamic memory”. Anomalous diffusion in crowded biological media was found experimentally in cellular membranes and cyto- and nucleoplasm [8]. In all of these cases, the chaotic behavior is responsible for ergodicity breaking and transport deceleration in comparison with the classical diffusion law.

Ergodicity breaking is also related to the creation of information and entropy production in a dynamical system. In an ergodic system, information (or memory about past states) is constantly erased, and therefore, entropy is created. The newly created information may result in the newly formed genome, which can be selected by macroevolution and further amplified into a larger population through microevolution. 

While ergodicity breaking in dynamical systems is associated with the chaotic trajectories in their configurational space, the concept of *genome chaos* has been suggested to explain rapid and uncontrollable evolution of malignant cancer cells [9,10,11,12]. Simple models with three competing cell populations (host, immune, and tumor cells) may result in complex chaotic behaviors [13,14]. Chaotic behavior can lead to a significantly higher maximum tumor size when compared to non-chaotic behaviors, since increasing the parameter associated with the killing of tumor cells by immune cells is demonstrated to increase the maximum tumor size, as modeled numerically [15]. Genetic instability explains the cell phenotype changes that take place during cancer progression [16]. Nikolov et al. [17] found that the growth and progression of tumors have features similar to “strange attractors” in the dynamical systems, combining local instabilities with global stability. Rocco et al. [18] suggested that the emergence of distinct growth phenotypes in clonal populations is related to weak ergodicity breaking. 

Heng et al. [12] proposed that karyotype-mediated macroevolution, rather than gene mutation, is the common driving force for most cancers. Their model implies a two-phased cancer evolution mechanism with genome alteration-mediated macroevolution, followed by gene mutation/epigenetic alteration-mediated microevolution. High stress induces genome chaos and macroevolution allows for new system survival, while microevolution aids system modification, providing an advantage for stepwise proliferation and competition [12].

**Figure 1:**
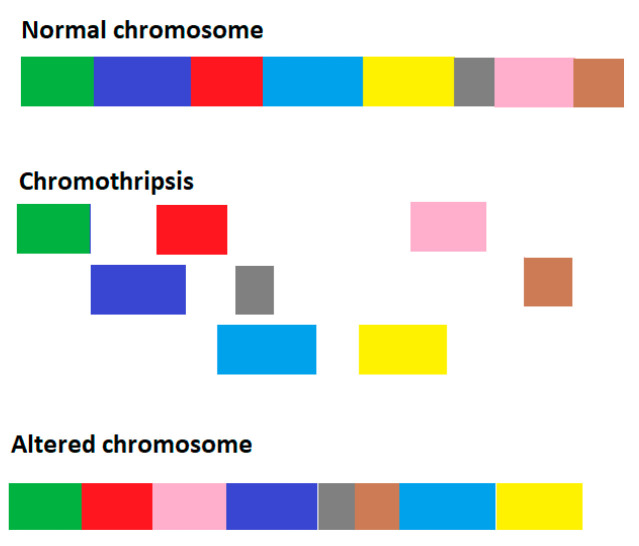
Schematic showing chromothripsis. Colors represent different sections of the chromosome.

One particular type of genome chaos is associated with *chromothripsis* [19], a massive genomic rearrangement, which is of particular interest for the formation of cancer. Chromothripsis is a catastrophic event when the chromosome is shattered into slices and then reassembled in a random order with mutation rates significantly increased (Figure 1).

Since the first observation of chromothripsis in the genome of a chronic lymphocytic leukemia, when dozens of chromosomal rearrangements were found in the long arm of chromosome 8 and several other chromosomes, similar patterns have been found in many human cancers including melanomas, sarcomas, colorectal, lung, and thyroid cancers, supratentorial ependymoma, chondromyxoid fibroma, Ewing sarcomas, and many other types [20]. Random mixing of chromosomal fragments is associated with ergodicity. 

In the present paper, we will suggest an analytical model for entropy creation and ergodicity during chromothripsis; we will also present a computational model for the mutation of the BCL2 gene responsible for apoptosis in cancer cells. The selection of the BCL2 gene was motivated by its significance as a crucial housekeeping gene and regulator of apoptosis in cancer cells and its critical role as a significant apoptosis inhibitor, often found in an overexpressed state across various cancer types. Additionally, it plays a pivotal role in the chromothripsis process observed during the transformation of cancer cells in the blast phase [21] (Brierley et al., 2023). It is well established that somatic cells undergo apoptosis in response to substantial DNA damage, often resulting from multiple double-stranded DNA breaks [22] (Shorokhova et al., 2021). The Bcl-2 gene alone does not singularly dictate the maintenance of clones at a 10% frequency. Rather, it is one of several important genes involved in this intricate process, including notable contributors like TP53 and RB genes [22] (Shorokhova et al., 2021). The antiapoptotic BCL-2 protein is important for the survival of rhabdomyosarcoma cells [23] (Heinicke, U. et al. (2018)).

In addition, we will review experimental data on time and population averages of cloned organotropic supermalignant RA-2 rhabdomyosarcomas of rat stem cells and investigate the possibility and experimental evidence that increasing the degree of chaos beyond the non-ergodic threshold may lead to the self-destruction of the tumor cells.

## 2. Materials and Methods

In this section, we present models for calculating entropy and ergodicity defects in the presence of chromothripsis as well as the rate of mutation of the BCL2 gene responsible for cancer cells’ apoptosis.

### 2.1. Model for Genome Chaos

In physics, chaos is defined as a property of a complex system whose behavior is unpredictable due to its great sensitivity to small changes in initial conditions. Usually, such systems are dynamically unstable, and small fluctuations grow exponentially with time. While the concept of genome chaos is widely used, there is no common definition of this term. A process of complex, rapid genome reorganization, caused by chromosomal instability and resulting in the formation of chaotic, unpredictable genomes, is usually implied by genome chaos [10,11,12].

We apply the concepts of ergodicity and entropy to genome chaos. First, we assume that the mutation rate *μ*(*θ*), defined as the probability of mutation per nucleotide per division, depends on a quantitative mutagenicity parameter, θ, characterizing the pressure from the environment increasing the probability of mutations.

During chromothripsis, up to thousands of chromosomal rearrangements can occur in a single event (Figure 1). Thus, if the original composition of an affected chromosome is $\left(G_{1},G_{2},\ldots G_{N}\right)$ , where *G_n_* is the *n*-th fragment of the chromosome, after shattering and consequent stitching, the sequence of genes can be rearranged in a random manner as $\left(G_{m_{1}},G_{m_{2}},\ldots G_{m_{N}}\right)$ . Some genomic information can be lost during the event. According to estimates, DNA breaks repaired by mitotic gene conversion are accompanied by surprisingly high mutation rates which are more than 1000-fold higher than spontaneous mutations [19]. The rate of spontaneous mutations in somatic human cells is about $\mu_{0}=1.4\times 10^{-10}\;$ nucleotides per cell per division [24]. In cancer cells without chromothripsis, such rate may be on the order of $\mu_{can}=10^{-7}$ [25].

The exact mechanisms of shattering and stitching remain obscure; however, there is growing evidence that the decrease in protein P53, which plays a central role in maintaining genome stability, correlates with chromothripsis. The corresponding gene, TP53, is the most frequently mutated gene in human cancer [26]. The appearance of chromosomal bridges and micronuclei in cells serves as a visual manifestation of chromothripsis. As far as the frequency of chromothripsis in cancer cells goes, in a recent survey of 4934 cancers, Zack et al. [27] suggested that chromothripsis occurred in 5% of all samples, with frequencies ranging from 0% in head and neck squamous carcinomas to a maximum of 16% in glioblastomas.

We now will distinguish three stages of the behavior of the system. At the first stage, no chromothripsis occurs. Following Rocco et al. [18], the cell state is viewed as a point in a multidimensional configuration space, characterized by gene expression profiles, $x_{n}$ , where the epigenetic landscape is a hypersurface formed by inverse probability for a cell to be at a certain state. The epigenetic landscape is analogous to the energy landscape of a Hamiltonian system, $H\left(x_{n}\right)$ . A phenotype is defined as the basin of attraction of each stable state (local minimum) on the landscape. The average barrier height between the stable states, ∆h, characterizes the probability of switching between the states (in other words, of a mutation) and can be estimated as $\Delta h=1/\mu_{0}=7\times 10^{9}$ . The average time that the system spends at a certain phenotype’s basin of attraction, *t*_0_, is larger than the observation time; for that reason, the system is essentially non-ergodic [18]. Essentially, the cells are in homeostasis.

In the second stage, massive rearrangements occur due to chromothripsis. Rearrangement of the fragments results in mixing, which can be estimated quantitatively using Shannon entropy [28]:

(1) $$S_{r}\left(N\right)=-N\ln\left(N\right)$$

For *N* chromosomal segments participating in the rearrangement, the number of rearrangement variants is *P*(*N*) = *N*! A simple statistical physics analogy can be a phase transition caused by increasing temperature above the melting point. The stability in a thermodynamic system at constant temperature and pressure is characterized by the Gibbs free energy defined as the difference between the enthalpic and entropic terms. The positive sign of the Gibbs energy change prohibits the spontaneous reaction:

(2) ΔG=Δh−θΔS>0

where the enthalpic term, ∆h, characterizes the resistance to mutation. Hence, $\theta_{0}=\frac{\Delta h}{\Delta S}$ . Note that the purpose of Equation (2) is to establish an analogy with the thermodynamics of phase transitions rather than to determine the exact value of the mutation rate at which chromothripsis occurs. This is because the quantitative characteristics would depend on the definition of the variable θ.

During chromothripsis, essential mixing of the chromosomal segments occurs. The degree of ergodicity can be estimated by calculating the ergodicity defect. For ideal mixing, the process is expected to be ergodic and the state of the cancer cells is not homeostatic.

At this stage, the mutation rate exceeds the critical threshold, $\mu >\mu_{cr}$ , so that mutations are too intense for cells to survive. As far as the biochemical mechanism goes, the gene that is responsible for apoptosis in cancer cells is BCL2 [29]. It is therefore hypothesized that when the critical number of mutations in cancer cells is achieved, apoptosis is induced.

Several measures of deviation from the ergodic behavior have been introduced in the literature to account for the non-ergodic behavior. Földes-Papp and Baumann [5] (2011) suggested decoupling the effects of the molecular crowding and the temporal heterogeneity by presenting the power exponent, which controls the dynamics of the interaction network, as a product of these two factors. Scott et al. [30] suggested the ergodicity defect *D*, defined at different scales (on a map T), with respect to a basis of functions *f* given by an integral of the square of space and time averages:

(3) $$D\left(f,T\right)\propto \int^{\;}{\left(f^{*}\left(x,T\right)-\bar{f}\right)}^{2}dx$$

where $f^{*}$ and $\bar{f}\;$ are the time and space averages.

### 2.2. Gene Mutation and Translation

The wild-type BCL2 gene sequence (ENST00000398117.1) was retrieved from the COSMIC database. A Python script was utilized to simulate genetic mutations with the following parameters: a mutation rate of 10^−5^ and about 86 cell divisions. The Python script simulates genetic mutations in a gene sequence using a Monte Carlo approach. The *mutate_gene* function introduces mutations based on a given mutation rate, altering nucleotides randomly. The main part of the script conducts a Monte Carlo simulation, repeatedly applying the mutation function to estimate the average number of iterations (divisions) required for a mutation to occur. After 1000 iterations, the average number of divisions was found to be 86.36.

### 2.3. Protein Stability Prediction

The wild-type (wt) structure, a component of the Bcl2-BINDI complex, was sourced from the Protein Data Bank as a crystal structure (PDB ID: 5JSN) with a resolution of 2.1 Å [31]. Predictions for mutant forms of the Bcl-2 protein were generated using the SWISS-MODEL algorithm [32] according to the protocol published elsewhere [28]. To assess protein stability, the standard Rosetta protocol was implemented, involving the calculation of the energy score (ES). This stability prediction method was executed by aligning root-mean-square-deviation (RMSD) values with Rosetta energy parameters, as outlined by Ramelot et al. [33]. The differences in Rosetta energy scores for mutated forms (Δ*ES*) were computed using the following equation:

(4) $$\Delta ES=ES_{mut}-ES_{wt}$$

where *ES_mut_* and *ES_wt_* are the Rosetta energy score values for the wild-type and mutated forms, respectively.

### 2.4. Protein Function Prediction

The crystal structure of Bcl-2 in association with a Bax BH3 peptide was retrieved from the Protein Data Bank (PDB ID: 2XA0) with a resolution of 2.7 Å [34] to determine the Bcl2-Bax binding site. The molecular interaction between the protein and the peptide was evaluated utilizing the ZDOCK molecular docking server for the prediction of protein–protein complexes and symmetric multimers [35]. The binding energy (*E_bind_*) was calculated by using the PPI-affinity tool designed to predict and optimize the binding affinity of protein–peptide and protein–protein complexes [36].

## 3. Results and Discussion

In this section, we present modeling results for mutations of the BCL2 gene as a main cancer proapoptotic inhibitor during chromothripsis and experimental observations on increasing genome chaos by selection [37,38,39].

### 3.1. Modeling Results for the Rate of Mutation of the BCL2 Gene

Before the stability and function of Bcl-2 were analyzed, the wild-type BCL2 gene (comprising 720 nucleotides) underwent a random mutation, resulting in a gene sequence with a Hamming distance of 1. This mutated sequence was subsequently translated into a protein sequence, which exhibited a sequence identity of 99.58% and a similarity score of 1277 when compared to the wild-type protein. A single nucleotide polymorphism (SNP) at amino acid position five was detected, with a G→W substitution.

**Figure 2:**
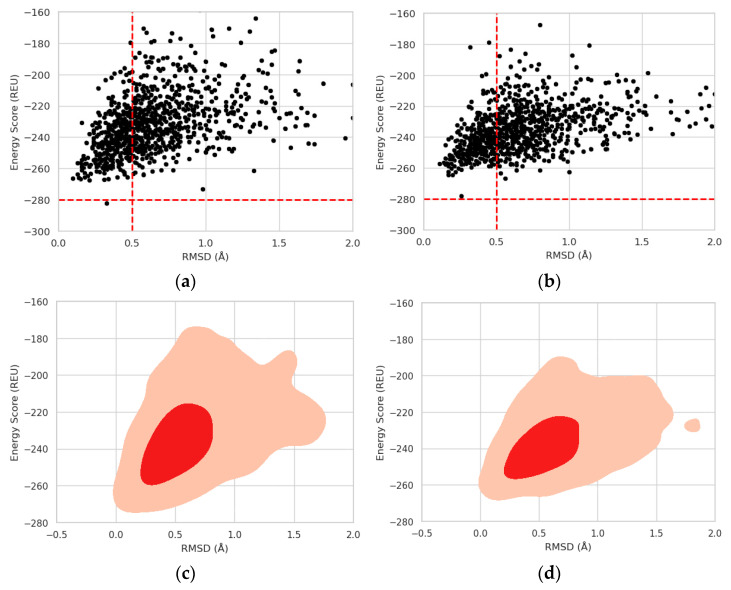
Energy score (ES) in Rosetta energy units (REU) vs. RMSD scatter (**a**,**b**) and contour plots (**c**,**d**) for the protein molecules including wild-type and mutants of Bcl-2. The energy thresholds (ES = −280 REU and RMSD = 0.5 Å) are depicted as dashed lines.

**Figure 3:**
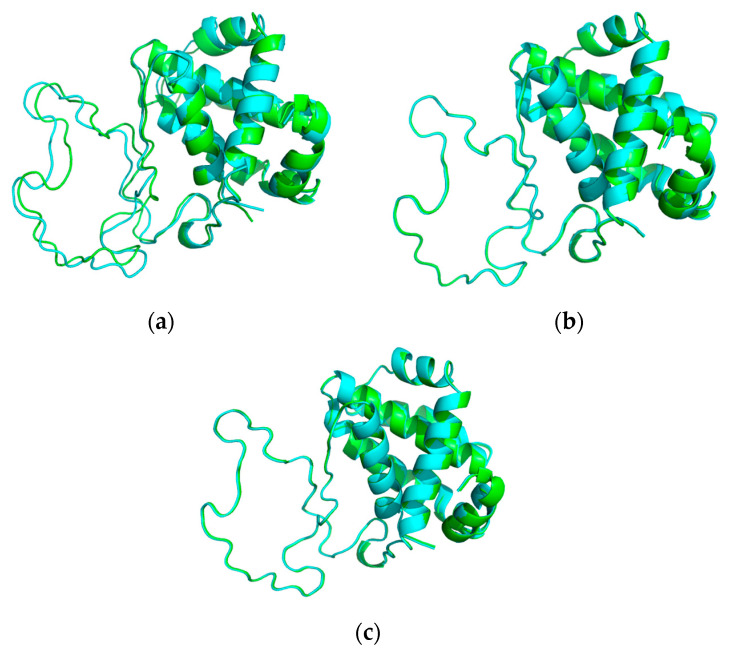
The 3D structural alignments of the wild-type and mutant template-based homology models (**a**), the wild-type homology model alongside its minimal structure determined by the Monte Carlo method (**b**), and the mutant homology model with its minimal structure determined by the Monte Carlo method (**c**).

Following this, a Rosetta protocol based on the Monte Carlo method identified a significant decrease in stability (Δ*ES* = 4.11 REU) for the mutated protein. The energy score (*ES_mut_*) for the G5W mutation was −278 REU, compared to −282.11 REU (*ES_wt_*) for the wild-type protein (refer to Figure 2a,b). The energy score for the mutated protein exceeded the energy threshold (*TS* = −280 REU), indicating the effect of the SNP. All minimum energy conformations were within the threshold range (0.5 Å) determined in our previous experiments for the molecular folding of the Trp-Cage protein [40,41]. Figure 3 presents the 3D structural alignments of the wild-type and mutant template-based homology models with an RMSD value of 0.46 Å, the wild-type homology model alongside its minimal structure with an RMSD value of 0.31 Å, and the mutant homology model and its minimal structure with an RMSD value of 0.27 Å.

**Figure 4:**
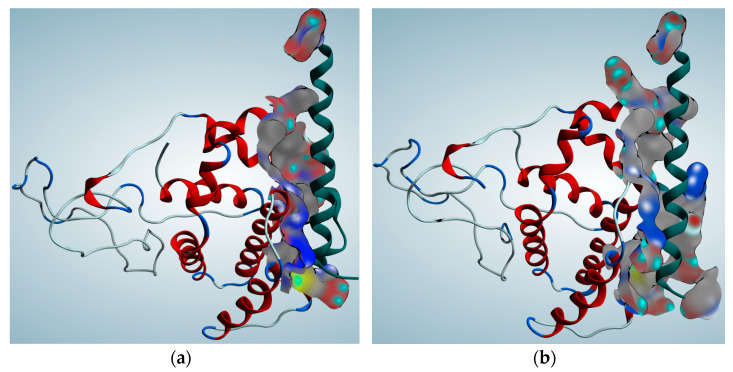
Predicted protein-peptide (Bcl2-BH3) interaction poses for (**a**) wild-type and (**b**) mutated protein calculated by the ZDOCK algorithm. The BH3 peptide is depicted in green. The molecular surface is implemented to visualize the protein–peptide binding site.

To evaluate the mutation’s impact on protein folding, the kernel density estimate was employed to compare energy score versus RMSD plots, which often form a triangular shape indicative of a protein folding pattern. The base of the triangle (lower RMSD values) represents near-native structures that have been refined and thus have lower energy scores. Consequently, the wild-type protein exhibited a well-formed triangle, while the mutated form displayed a distorted folding pattern, suggesting the mutation’s influence on protein folding (Figure 2c,d). Finally, the interaction between Bcl-2 and a Bax BH3 peptide was assessed to determine the impact of the SNP on the inhibition of apoptosis in the cancer cell. The results indicated that the Bcl-2-Bax interaction (Figure 4a,b) was impaired by the point mutation due to an increase in the binding energy for the mutated form (*E_bind_* = −11.1 kal/mol) compared to the wild-type (*E*_bind_ = −11.3 kcal/mol).

### 3.2. Case Study: Self-Destruction of Rhabdomyosarcoma (RA) of Rats

**Figure 5:**
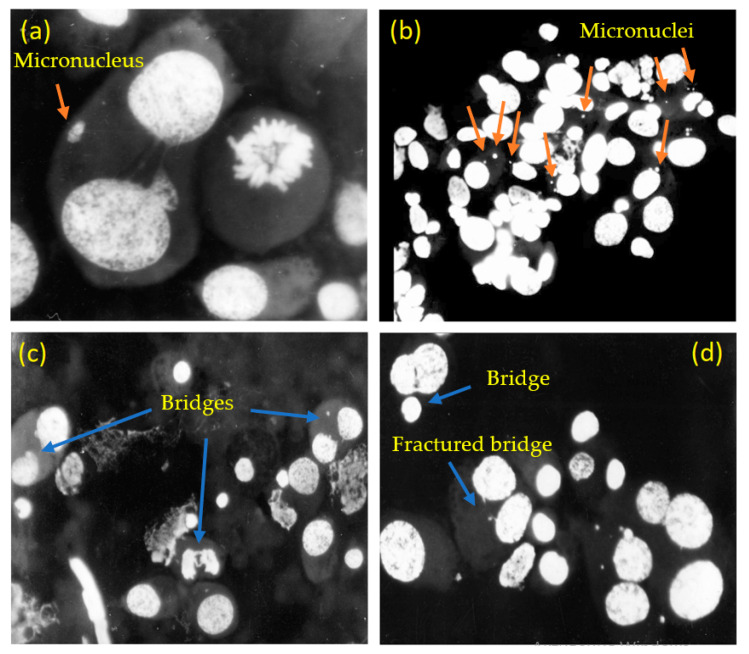
Bridges and micronuclei in RA-2 transplantable rhabdomyosarcoma cells of rats. (**a**) A micronucleus at magnification ×1000. (**b**) Numerous cells with micronuclei (some marked with orange arrows) at magnification ×200. (**c**) Cells with bridges (blue arrows) at magnification ×200 and (**d**) ×400.

Already in the 1990s, Kravtsov and co-workers studied transplantable rhabdomyosarcoma (RA) cells from rats induced by 20-methylcholanthrene in lung tissue. They conducted several stages of selection for increasing genome chaos [37,38,39], applying the methodology that had been earlier developed in Ref. [42]. The frequency of cells with micronuclei (FCM) was used as a marker of genome chaos. Experimental metastases clones of RA-2 had a spherical shape with dimensions of 3–5 mm. Micronuclei were usually observed in single-nucleus cells in interphase, as evidenced by their external morphological features. The size of the observed micronuclei varied from 1 to 3 μm; the results are based on Ref. [38] (Figure 5).

Artificial selection of cell clones with a high degree of spontaneous karyotype instability was used. Clones 3–5 mm in diameter prepared from the lungs of grafted rats were cut in half; one half of each clone was used to prepare a smear, and the other half of the clone was placed in a vial with medium 199. As a rule, the duration of the determination of FCM for a sample of 50–60 clones did not exceed 6 h. The selection for increasing FCM was effective and led to a gradual increase in FCM to 6.9–8.1% (Table 1). After three to four steps of selection for an increase in FCM, more than 25% of the clones had an FCM greater than 9.0%. The average value of the FCM was 6.9%, and the range of variability was from 0.5 to 17.0%.

Metastases clones stopped their proliferation and were subjected to apoptosis at the average FCM, close to 4.7%, and the maximum FCM was close to 15%, roughly speaking, on the order of 10%.

### 3.3. Calculation of Ergodicity Defect

Chromothripsis increases the rate of mutation of cancer cells by three orders of magnitude, leading to the rates of $\mu_{c}\sim 10^{-4}\;$ mutations per division per nucleotide. If in the population of *n* cancer cells the fraction of cells with chromothripsis constitutes *f_c_*, the average mutation rate is given by the following equation:

(5) $$\mu =f_{c}\mu_{c}+\left(1-f_{c}\right)\mu_{can}$$

Assuming the values of $f_{c}=0.1$ corresponding to 10% FCM, $\mu_{c}\sim 10^{-4}$ and $\mu_{can}\sim 10^{-7},$ yields $\mu =1.009\times 10^{-5}$ mutations per division per nucleotide.

The pre-selection distribution of chromothripsis cells (minimum FCM = 0%, average FCM = 0.6%, and maximum FCM = 2.0%) is assumed to be an equilibrium distribution. The population of cells maintains this distribution throughout the generations of division. Therefore, the time average (generation average) value is equal to the population average and the population is ergodic. In other words, the clone average value does not change with time and therefore coincides with the time average. However, during the selection of cells for increasing chromothripsis, the ergodic equilibrium is shifted away from the ergodic state. The non-ergodic state eventually leads to the destruction of all cancer cells by apoptosis.

**Figure 6:**
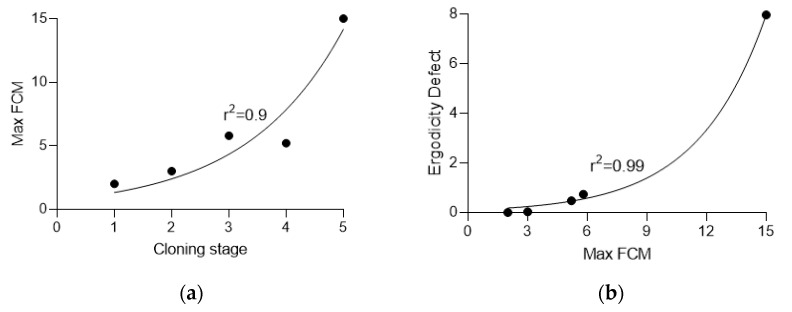
Dependency of the (**a**) maximum frequency of cells with micronuclei (FCM) on the selection stage and (**b**) ergodicity defect on the maximum FCM.

The effective mutation rate was calculated with Equation (5), and the ergodicity defect was calculated using Equation (3). The values are presented in Table 1 and Figure 6.

The data suggest that during the selection process, the number of cells affected by chromothripsis grows quickly, reaching maximum possible values at the fifth stage, or even earlier in some experiments [37,38,39]. Increasing the frequency of cells with micronuclei (i.e., likely subject to chromothripsis) results in a sharp increase in the ergodicity defect. The process continues until the concentration of FCM reaches 10–15% of total cancel cells.

The rationale behind our choice of the Bcl-2 gene as a focal point in our investigation is rooted in its dual significance as a crucial housekeeping gene and a key regulator of apoptosis in cancer cells. Furthermore, its pivotal role extends to the chromothripsis process observed during the transformative blast phase of cancer cells, as highlighted by Brierley et al. [21]. As established in the literature, somatic cells respond to substantial DNA damage by undergoing apoptosis, a process often triggered by multiple double-stranded DNA breaks [22]. The integral role in a network of essential genes governing this intricate process includes the TP53 and RB genes, as elucidated by Shorokhova et al. [22]. However, modern molecular modeling techniques have the limitations in capturing the complexity of a chromothripsis event. Therefore, we chose the Bcl-2 gene as an example to elucidate the threshold concentration of cells with chromothripsis, correlating it with the deterioration of Bcl-2 or similar proteins responsible for genomic stability. Furthermore, the significance of the antiapoptotic BCL-2 protein extends to its importance in the survival of rhabdomyosarcoma cells, as highlighted by Heinicke et al. [23] in their study on the BCL-2 selective inhibitor ABT-199. This study demonstrates how the selective inhibition of BCL-2 primes rhabdomyosarcoma cells for apoptosis induced by histone deacetylase inhibitors, shedding light on potential therapeutic avenues.

Modeling the BCL2 gene mutation for 86 generations at $\mu =10^{-5}$ mutations per division per nucleotide yields the overall probability of mutation on the order of *p* = 1.

We therefore conclude that the threshold value of the concentration of cells with chromothripsis corresponding to the deterioration of Bcl2 or a similar protein responsible for genomic stability is on the order of $f_{c}=0.1$ (or 10%). This may explain the observation that RA-2 cells tend to reach the maximum concentration of cells with chromothripsis before being subjected to apoptosis. The observed phenomenon provides potential new ways of cancer treatment by inducing tumor cells’ apoptosis.

**Figure 7:**
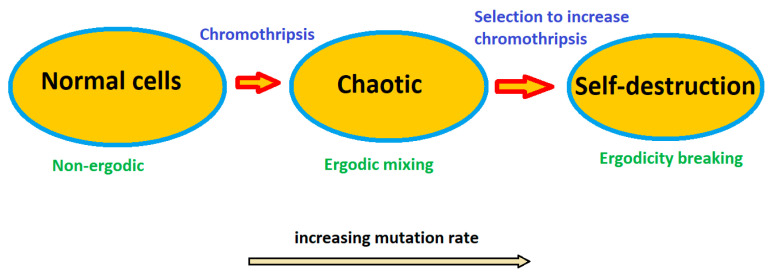
Chromothripsis marks the transition to ergodic behavior, while further increasing the mutation rate results in the apoptosis of malicious cells. Note that increasing the degree of chaos beyond the non-ergodic threshold may also lead to the emergence of clones that are resistant to the treatment methods.

The transition to genome chaos leads to ergodic mixing of genome segments, whereas the further increase in the mutation rate results in the death of the cancer cells (Figure 7). To some extent, a physical analogy can be suggested with the transition from a solid to liquid state of matter (with a chaotic motion of molecules) and then to evaporation with increasing temperature. The increasing mutation rate plays the role of the increasing temperature in this case. Note also that despite the fact that induced genome chaos can eliminate most cancer cells, caution is needed, as treatment-induced genome chaos may also lead to rapid drug resistance [43].

## 4. Conclusions

Genome chaos is associated with cancer propagation, at least for some types of cancer. Genomic chaos-mediated genomic rearrangement not only increases the mutation rate but also gives rise to a new system-level coding, defining the newly formed system. Chromothripsis is one of the most prominent manifestations of genome chaos. During chromothripsis, mixing of the chromosomal segments occurs, and therefore, entropy is created while information is erased. The regime with erased information, i.e., without memory, is ergodic. However, with the increasing rate of mutations, malignant cells deviate from the ergodic regime, and eventually, apoptosis happens. Computational modeling of the mutations in the BCL2 gene responsible for apoptosis in tumor cells shows that the probability of mutations approaches the unity at the concentration of the cells with chromothripsis on the order of 10%. This is consistent with experimental data on cloned rhabdomyosarcomas from rat stem cells, artificially selected for increasing the rate of genome chaos. Clones with a frequency of chromothripsis above 10% result in cell apoptosis, possibly due to mutations in the BCL2 gene. Potentially, this can be used for suppressing cancer cells by stimulating increased mutation rates and shifting them into a non-ergodic proliferation regime, although the possibility of drug resistance should be considered.

## Figures and Tables

**Table 1:** Changes in observed minimum, average, and maximum FCM and calculated effective mutation rate and ergodicity defect at different stages of the selection for increasing FCM.

Stage	Clones	Min FCM	Average FCM	Max FCM	Effective Mutation Rate, ×10^−4^	Ergodicity Defect
0	48	0.0	0.6	2.0	6.99	0
1	50	0.0	0.8	3.0	8.99	0.03
2	52	0.0	1.7	5.8	17.98	0.74
3	48	0.0	1.6	5.2	16.98	0.48
4	47	1.0	4.7	15.0	47.95	7.96

## Data Availability

Publicly available datasets were analyzed in this study. These data can be found here: https://github.com/virtualscreenlab/Genome-Chaos accessed on 1 December 2023.
